# Focal Vibration Therapy for Motor Deficits and Spasticity Management in Post-Stroke Rehabilitation

**DOI:** 10.3390/brainsci14111060

**Published:** 2024-10-25

**Authors:** Federica Giorgi, Danilo Donati, Daniela Platano, Roberto Tedeschi

**Affiliations:** 1 Pediatric Physical Medicine and Rehabilitation Unit, IRCCS Institute of Neurological Sciences, Via Zamboni 33, 40126 Bologna, Italy; 2 Physical Therapy and Rehabilitation Unit, Policlinico di Modena, 41125 Modena, Italy; 3 Clinical and Experimental Medicine PhD Program, University of Modena and Reggio Emilia, 41125 Modena, Italy; 4 Department of Biomedical and Neuromotor Sciences, Alma Mater Studiorum, University of Bologna, 40127 Bologna, Italy; 5 Physical Medicine and Rehabilitation Unit, IRCCS Istituto Ortopedico Rizzoli, 40136 Bologna, Italy

**Keywords:** focal vibration therapy, stroke rehabilitation, spasticity reduction, motor function recovery, pain management

## Abstract

Background: Focal mechanical vibration therapy has gained attention as a potential intervention to improve motor function while decreasing spasticity and pain in post-stroke patients. Despite promising results, there remains variability in study designs and outcomes, warranting a review of its clinical efficacy. Methods: A review was conducted to evaluate randomized controlled trials (RCTs) investigating the effects of focal mechanical vibration therapy on post-stroke rehabilitation. Six studies were included, assessing outcomes such as spasticity reduction (using the Modified Ashworth Scale), motor function recovery (Wolf Motor Function Test, Fugl-Meyer Assessment), and pain management (Visual Analog Scale, Numerical Rating Scale). The quality of studies was evaluated using the PEDro scale and RoB-2 tool. An overview review was conducted to provide a comprehensive analysis of the topic. Results: The included studies demonstrated significant reductions in spasticity and improvements in motor function in most patients receiving focal vibration therapy. Notable improvements were observed when focal vibration was combined with other rehabilitation techniques, such as progressive modular rebalancing or robotic rehabilitation. Pain levels were also reduced in several studies. However, differences in vibration parameters (frequency, amplitude), small sample sizes, and short follow-up periods limit the generalizability of the findings. Conclusions: Focal mechanical vibration therapy appears to be an effective adjunct in post-stroke rehabilitation, particularly for reducing spasticity and improving motor function. Although short-term benefits are promising, further research is required to determine long-term efficacy and optimal treatment parameters. This review evaluates the effectiveness of focal vibration therapy in treating motor deficits and spasticity in post-stroke patients. The results suggest its potential to improve these conditions, though further studies with larger sample sizes are needed to confirm its long-term efficacy.

## 1. Introduction

Stroke is a sudden cerebrovascular event that results in neurological deficits, often requiring extensive and complex rehabilitation [1,2,3]. Stroke is one of the leading causes of disability worldwide, with approximately 80% of stroke survivors experiencing motor deficits, including spasticity. These motor impairments significantly affect functional independence and quality of life. The economic burden associated with stroke rehabilitation is substantial, with an estimated annual direct and indirect cost of €45 billion in Europe alone, which includes costs related to long-term healthcare and loss of productivity due to disability [4,5].The majority of stroke survivors face significant challenges, including complications that threaten their survival and impair their independence [6]. These complications are multifaceted, ranging from motor deficits to cognitive impairments, often leaving patients with long-term disabilities. In recent years, focal mechanical vibration therapy (FMVT) has emerged as a promising intervention in neurorehabilitation [7,8,9,10]. This therapy, known for its versatility, can be used across various clinical contexts, producing different therapeutic effects depending on the parameters set. FMVT acts by stimulating neuromuscular receptors such as muscle spindles and Golgi tendon organs, which transmit signals to the central nervous system. These mechanical oscillations can enhance neuroplasticity, a key mechanism in the recovery process post-stroke. By modulating the excitability of the spinal cord and cortex, FMVT promotes improved motor output, aiding in the reduction of spasticity and enhancement of motor control. Evidence suggests that early intervention during the subacute phase when neuroplasticity is at its peak, can optimize these benefits [11]. The primary outcomes of FMVT include enhanced muscle recruitment and the reduction of spasticity, which are both crucial in the recovery process of stroke patients. The idea for this review stems from the increasing interest in non-pharmacological therapies that could complement traditional rehabilitation approaches for stroke patients. FMVT acts not only at the muscular level but also influences the central nervous system, promoting neuroplasticity—a process through which the brain reorganizes itself in response to injury [12,13,14,15,16,17,18,19,20]. This ability to influence both peripheral and central systems makes FMVT an intriguing option in the rehabilitation landscape. Despite its potential, there remains a lack of consensus on the optimal application of this therapy in post-stroke recovery [21,22,23,24,25,26,27,28]. Therefore, it is necessary to review the available literature to determine the effectiveness of FMVT in improving outcomes for stroke patients. The research question that guides this review is whether focal vibration therapy can be effectively integrated into the rehabilitation protocols for patients with post-stroke sequelae. Stroke rehabilitation is traditionally centered on motor recovery, with a strong emphasis on improving functional independence. However, spasticity, pain, and muscle weakness are common barriers to successful rehabilitation [29,30,31,32,33,34]. These factors complicate the recovery process, leading to prolonged disability and reduced quality of life. Given these challenges, it is imperative to explore therapies that target these specific issues in stroke patients. FMVT, by modulating neural and muscular responses, could offer a targeted approach to addressing spasticity, enhancing motor control, and reducing pain [35,36,37,38,39,40,41,42,43,44]. These effects are particularly relevant in stroke rehabilitation, where motor function recovery is a primary goal. FMVT works by delivering mechanical oscillations to specific muscles or tendons, activating neuromuscular receptors such as muscle spindles and Golgi tendon organs [37]. These receptors, in turn, transmit signals to the central nervous system, influencing both sensory and motor pathways. Studies suggest that these vibrations can alter the excitability of the spinal cord and cortex, leading to improved motor output and decreased muscle stiffness [16,17]. The versatility of the therapy allows it to be used in various settings, including sports medicine, post-surgical rehabilitation, and notably, neurological disorders such as stroke. In the context of stroke rehabilitation, focal vibration has shown potential in reducing spasticity—a common and often debilitating condition in stroke survivors. Spasticity results from damage to the motor neurons, leading to increased muscle tone and involuntary muscle contractions, which severely impair mobility [45,46]. By reducing spasticity, FMVT may enhance patients’ ability to engage in rehabilitation exercises and improve their overall functional outcomes. Despite these promising mechanisms, the clinical evidence supporting the use of focal vibration in stroke rehabilitation is still emerging. Various studies have examined its effects on muscle function, spasticity, and pain, but the results have been inconsistent. Some studies report significant improvements in motor recovery and spasticity reduction, while others show only modest or short-term benefits [45]. This discrepancy may be due to differences in study design, patient populations, or the specific parameters used for the FMVT. Therefore, it is crucial to critically examine the existing research to determine the overall efficacy of this intervention. This review aims to consolidate the current evidence on FMVT in post-stroke rehabilitation. By analyzing studies that evaluate its effects on spasticity, motor function, and pain, this review seeks to clarify whether FMVT should be integrated into standard rehabilitation practices for stroke survivors. Although this review is not systematic, it intends to provide a comprehensive overview of the topic, highlighting areas where FMVT has shown promise and identifying gaps in the literature that warrant further investigation. The ultimate goal is to assess whether this therapeutic approach can contribute to better outcomes in stroke rehabilitation, thereby improving the quality of life for stroke survivors.

## 2. Materials and Methods

This review adhered to the methodological framework outlined by the Joanna Briggs Institute (JBI) [47] for conducting scoping reviews. Reporting was guided by the PRISMA-ScR (Preferred Reporting Items for Systematic reviews and Meta-Analyses extension for Scoping Reviews) [48] Checklist.

The study adhered to predefined eligibility criteria for inclusion and exclusion, focusing on adult patients with confirmed stroke presenting motor deficits or spasticity. Data extraction was structured using a standardized form to capture study characteristics, patient demographics, and outcomes. The methodology was clearly mapped out to ensure replicability and transparency.

### 2.1. Review Question

We formulated the following research question: “Is focal mechanical vibration therapy effective in improving spasticity, motor function, and pain management in patients with post-stroke sequelae, and should it be integrated into standard rehabilitation protocols”?

### 2.2. Eligibility Criteria

Studies were eligible for inclusion if they met the following Population, Concept, and Context (PCC) criteria.

Population (P): The target population included adults (18 years or older) who had experienced a stroke, either ischemic or hemorrhagic, with post-stroke sequelae such as motor impairments, spasticity, or chronic pain.

Patients included in the studies should have been diagnosed with stroke sequelae for at least one month or longer, ensuring the focus was on rehabilitation of chronic or subacute post-stroke conditions, rather than acute stroke management.

Both male and female participants of any ethnic background were eligible, as long as they presented with stroke-related motor deficits or neurological impairments, such as spasticity, muscle weakness, or difficulties with motor control.

Exclusion criteria typically involved patients with significant cognitive impairments, such as dementia, that might prevent them from following rehabilitation instructions, or those with severe comorbidities that could affect rehabilitation outcomes (e.g., advanced cardiovascular disease or uncontrolled diabetes).

Concept (C): The primary concept of interest was the application of focal mechanical vibration therapy. This therapy involves delivering targeted vibrations to specific muscle groups or tendons, aiming to influence neuromuscular control, reduce spasticity, improve motor function, and potentially alleviate chronic pain.

Studies were required to involve interventions where focal mechanical vibrations were applied either alone or in conjunction with other standard rehabilitation therapies. The vibration therapy could be delivered through a range of devices, provided they produced targeted mechanical oscillations at specific frequencies and amplitudes intended to influence muscle and neural activity.

The review specifically looked for studies evaluating the effectiveness of focal vibration in improving outcomes such as muscle spasticity, motor function (e.g., movement coordination, strength, range of motion), and pain management in stroke patients.

Context (C): The context for the studies included rehabilitation settings, which could vary from in-patient rehabilitation centers, outpatient clinics, or home-based rehabilitation programs, provided the focal vibration therapy was part of a structured rehabilitation plan.

Studies could be conducted in any country or healthcare system, reflecting different rehabilitation practices, as long as they focused on the use of focal mechanical vibration therapy in stroke patients.

The timeframe of rehabilitation varied, including both short-term (weeks to months) and long-term studies (several months to years), to assess the sustained effects of focal vibration on stroke recovery.

### 2.3. Exclusion Criteria

Studies that did not align with the specified Population, Concept, and Context (PCC) criteria were excluded from the review.

### 2.4. Search Strategy

An initial focused search was conducted in MEDLINE using the PubMed platform to locate relevant articles. The keywords and index terms identified were then utilized to create a comprehensive search strategy for MEDLINE. This strategy was subsequently adapted for use in other databases, including Cochrane Central, Scopus, and PEDro. Additionally, grey literature and reference lists from pertinent studies were also reviewed. The searches were carried out on 31 August 2024, without any date restrictions.

PubMed: (focal vibration OR mechano-acoustic vibration OR mechanical vibration) AND stroke AND (rehabilitation OR spasticity OR motor function OR pain).

Scopus: TITLE-ABS-KEY (“focal vibration” OR “mechano-acoustic vibration” OR “local vibration”) AND TITLE-ABS-KEY (stroke) AND TITLE-ABS-KEY (rehabilitation OR spasticity OR motor recovery OR pain).

Web of Science: TS = (“focal vibration” OR “mechano-acoustic vibration” OR “mechanical vibration”) AND TS = (stroke) AND TS = (rehabilitation OR spasticity OR motor recovery OR pain).

Cochrane: (focal vibration OR mechano-acoustic vibration OR mechanical vibration) AND stroke AND (rehabilitation OR spasticity OR motor recovery OR pain).

Pedro: (focal vibration) AND (stroke).

### 2.5. Study Selection

The study selection process employed a systematic approach suitable for a scoping review. Initially, search results were gathered and refined using Zotero, ensuring duplicates were removed. The screening was conducted in two stages: first, a review of titles and abstracts, followed by a detailed assessment of full texts. Both stages were carried out independently by two reviewers, with any disagreements resolved by a third reviewer. The process followed the PRISMA 2020 guidelines to maintain transparency and accuracy. This methodical approach was designed to identify articles that were directly relevant to the research question, ensuring a thorough and structured review process.

### 2.6. Data Extraction and Data Synthesis

Data extraction for the scoping review was carried out using a template based on the JBI tool, recording essential information such as authorship, country and year of publication, study design, patient characteristics, outcomes, interventions, procedures, and other relevant details. Descriptive analyses were performed, and the findings were presented numerically to illustrate the distribution of studies. The review process was thoroughly documented to ensure transparency, and the data were organized into tables for straightforward comparison and interpretation of the key aspects and results of the studies.

## 3. Results

**Figure 1:**
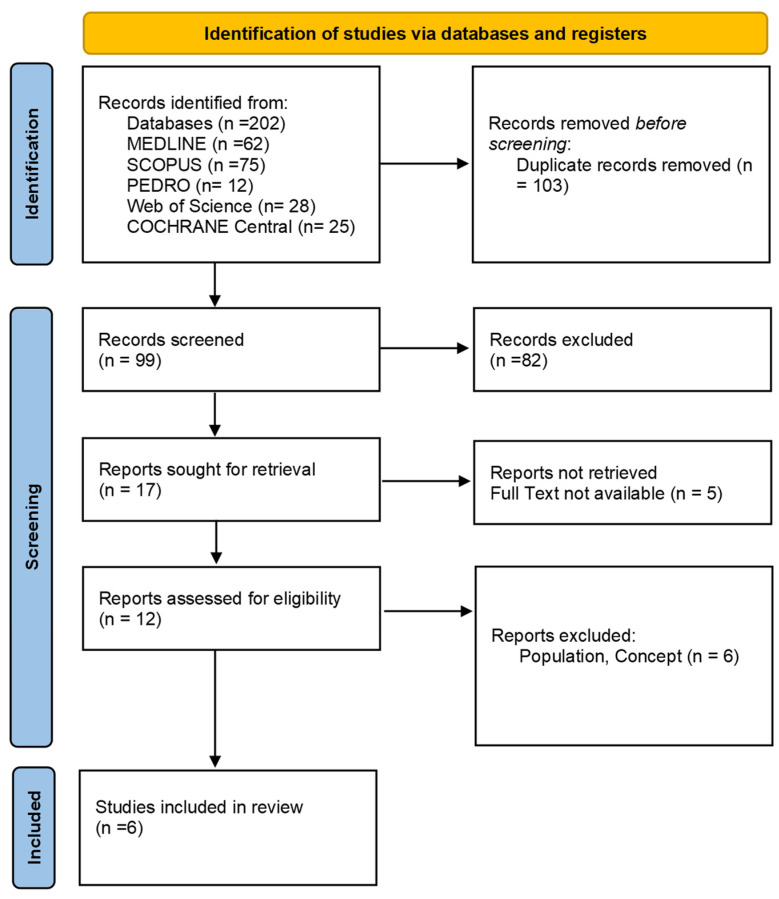
Preferred reporting items for systematic reviews and meta-analyses 2020 (PRISMA) flow diagram.

As presented in the PRISMA 2020-flow diagram (Figure 1), from 202 records identified by the initial literature searches, 196 were excluded, and six articles were included (Table 1 and Table 2). The quality of the studies was assessed with a PEDro scale and ROB2 (Table 2).

### 3.1. Spasticity Reduction

Spasticity, a common complication in post-stroke patients, was a primary outcome in most studies. The Modified Ashworth Scale (MAS) was predominantly used to measure spasticity levels.

- Chen Y.L. et al. (2022) [45]: A significant reduction in spasticity was observed in the groups receiving focal vibration (FV_GM and FV_TA) compared to the control group. The focal vibration groups had better outcomes in reducing ankle spasticity, particularly in the gastrocnemius muscle, with lower MAS scores post-treatment.
- Celletti C. et al. (2017) [15]: Spasticity reduction was observed across all groups, but the FMV + RMP group showed the most significant decrease in MAS scores. This suggests that combining focal vibration with progressive modular rebalancing (RMP) may enhance the effects of spasticity reduction.
- Toscano M. et al. (2019) [16]: Patients treated with rMV (repeated muscle vibration) demonstrated significantly lower spasticity levels compared to the sham group, with a marked improvement in MAS scores.
- Caliandro P. et al. (2012) [17]: While the intervention led to improved upper limb functionality, no significant differences were found in MAS scores for spasticity reduction compared to the control group.
- Costantino C. et al. (2017) [18]: The study group receiving local vibration treatment showed a significant reduction in upper limb spasticity, as evidenced by a marked decrease in MAS scores. This effect was absent in the control group.
- Calabrò R.S. et al. (2017) [19]: Significant spasticity reduction was reported in the study group receiving combined focal vibration and robotic rehabilitation. The MAS scores decreased by at least one point, indicating a substantial reduction in muscle stiffness.

### 3.2. Motor Function and Functional Recovery

Motor recovery was measured using various functional assessments, including the Wolf Motor Function Test (WMFT), Fugl-Meyer Assessment (FMA), and Functional Ambulation Classification (FAC).

- Chen Y.L. et al. (2022) [45]: Improvement in walking function was most evident in the control group that received Bobath therapy, as measured by the FAC score. Although the FV_GM group showed significant reductions in spasticity, motor function improvements were more pronounced in the conventional therapy group.
- Celletti C. et al. (2017) [15]: The FMV + RMP group had the most significant improvement in upper limb functionality, as measured by the WMFT. Both FMV + RMP and FMV + CP groups showed notable gains in motor function, suggesting that focal vibration can enhance muscle performance when combined with rebalancing techniques.
- Toscano M. et al. (2019) [16]: The study group exhibited significant improvements in motor recovery, with higher Fugl-Meyer scores compared to the control group. The Motricity Index (MI) also showed marked improvement, suggesting that focal vibration can contribute to enhanced motor function recovery.
- Caliandro P. et al. (2012) [17]: A significant improvement in upper limb function was observed in the intervention group, as measured by the WMFT. The effect was specific to the group receiving focal vibration, with a clear difference in functional recovery compared to the placebo group.
- Costantino C. et al. (2017) [18]: Grip strength improved significantly in the study group compared to the control group. This was measured using the Hand Grip Strength Test, indicating that focal vibration therapy enhanced muscle strength in both the affected and unaffected limbs.
- Calabrò R.S. et al. (2017) [19]: Patients receiving combined focal vibration and robotic rehabilitation exhibited significant motor function recovery. Fugl-Meyer scores showed marked improvement, and functional gains were sustained even 4 weeks post-treatment, suggesting the long-term benefits of the intervention.

### 3.3. Pain Reduction

Pain reduction, where applicable, was primarily measured using the Visual Analog Scale (VAS) or Numerical Rating Scale (NRS).

- Celletti C. et al. (2017) [15]: Pain reduction was significant in both the FMV + RMP and FMV + CP groups. VAS scores indicated a marked decrease in reported pain, especially in patients who received focal vibration in combination with other therapeutic exercises.
- Caliandro P. et al. (2012) [17]: No significant reduction in pain was observed in the intervention group. VAS scores did not show a clear difference between the groups, suggesting that focal vibration may not have had a substantial impact on pain in this particular study.
- Costantino C. et al. (2017) [18]: Pain levels, measured using the NRS, decreased significantly in the group receiving focal vibration therapy. This reduction was observed not only in the treated upper limb but also in the contralateral limb, suggesting systemic benefits of the intervention.

### 3.4. Cortical and Spinal Circuit Modulation

Some studies investigated the neurophysiological effects of focal vibration, particularly its impact on cortical excitability and spinal circuits.

- Calabrò R.S. et al. (2017) [19]: This study reported significant reductions in cortical excitability, as measured by short-interval cortical inhibition (SICI), and in spinal motor reflex activity (Hoffman’s Reflex, HMR). These changes correlated with improvements in spasticity and motor function, indicating that focal vibration can modulate both cortical and spinal circuits to facilitate recovery.

### 3.5. Secondary Outcomes (Mood, Disability, Anxiety)

Secondary outcomes such as disability levels, mood, and anxiety were measured using scales like the QuickDASH, Functional Independence Measure (FIM), Hamilton Rating Scale for Depression (HRS-D), and Hamilton Rating Scale for Anxiety (HRS-A).

- Costantino C. et al. (2017) [18]: Disability levels, as measured by QuickDASH, showed significant improvement in the study group compared to the control group. This was supported by improvements in FIM scores, indicating better functional independence in daily activities.
- Calabrò R.S. et al. (2017) [19]: The study group showed significant improvements in mood and reductions in anxiety. HRS-D and HRS-A scores demonstrated a marked reduction in depressive and anxious symptoms, which likely contributed to enhanced engagement in rehabilitation activities.

Table 2 provides a summary of the PEDro scores for each study, reflecting the methodological quality, and a detailed assessment of bias based on the RoB-2 tool across key domains (randomization, adherence to intervention, missing data, outcome measurement, and selective reporting). A higher PEDro score indicates a stronger study design, while the RoB-2 assessment categorizes the potential risk of bias as low or with some concerns in the various domains evaluated.

**Table 2:** Quality Assessment Using PEDro and RoB-2 Scales.

Study	PEDro Score	RoB-2 Assessment
Chen Y.L. et al. (2022) [45]	08/10.	Randomization Bias: Low (randomized allocation reported). Deviations from Intended Interventions: Low (adherence to intervention verified). Missing Outcome Data: Low (complete data for all participants). Measurement Bias: Low (objective measures like MAS and Clonus Test used). Selective Reporting Bias: Low (all pre-specified outcomes reported).
Celletti C. et al. (2017) [15]	07/10.	Randomization Bias: Low (adequate randomization procedure). Deviations from Intended Interventions: Low (well-described adherence and blinding). Missing Outcome Data: Low (minimal loss of follow-up). Measurement Bias: Low (objective scales like WMFT and MAS used). Selective Reporting Bias: Low (pre-registered protocol and all outcomes reported).
Toscano M. et al. (2019) [16]	07/10.	Randomization Bias: Low (random allocation clearly stated). Deviations from Intended Interventions: Low (adherence to protocol with sham control). Missing Outcome Data: Low (all participants accounted for). Measurement Bias: Low (validated outcome measures like NIHSS and Fugl-Meyer). Selective Reporting Bias: Low (full outcome reporting, no omissions).
Caliandro P. et al. (2012) [17]	06/10.	Randomization Bias: Low (adequate randomization). Deviations from Intended Interventions: Low (minimal deviation from protocol). Missing Outcome Data: Some concerns (minor loss of follow-up, but impact negligible). Measurement Bias: Low (objective measures like WMFT and MAS). Selective Reporting Bias: Low (outcomes reported as per trial protocol).
Costantino C. et al. (2017) [18]	08/10.	Randomization Bias: Low (randomization clearly described). Deviations from Intended Interventions: Low (proper blinding and adherence to protocol). Missing Outcome Data: Low (complete data reported). Measurement Bias: Low (objective assessments like Hand Grip Strength and MAS). Selective Reporting Bias: Low (all outcomes fully reported).
Calabrò R.S. et al. (2017) [19]	08/10.	Randomization Bias: Low (clear description of randomization). Deviations from Intended Interventions: Low (clear protocol adherence, no major deviations). Missing Outcome Data: Low (complete dataset, no missing data). Measurement Bias: Low (objective outcome measures used). Selective Reporting Bias: Low (outcomes pre-specified and fully reported).

Legend: PEDro Score: Physiotherapy Evidence Database Score, RoB-2: Risk of Bias 2 Tool.

## 4. Discussion

This review aimed to evaluate the efficacy of focal mechanical vibration therapy in post-stroke rehabilitation, particularly in reducing spasticity, improving motor function, and managing pain. The results, derived from six randomized controlled trials (RCTs), suggest that focal vibration therapy can indeed play a significant role in enhancing outcomes in post-stroke patients. However, several factors and limitations warrant careful consideration when interpreting these findings. The consistent reduction in spasticity observed across most studies underscores the potential of focal vibration therapy as a valuable intervention in post-stroke care. Spasticity, a debilitating consequence of stroke, often impedes functional recovery by limiting range of motion and increasing muscle stiffness. Studies by Chen Y.L. et al. (2022) [45] and Costantino C. et al. (2017) [18] demonstrated significant reductions in spasticity, as measured by the Modified Ashworth Scale (MAS). These findings align with the physiological mechanism of action of focal vibration, which modulates both peripheral and central neural circuits, thus reducing hyperexcitability in spastic muscles. Importantly, these effects were observed not only in the target muscles but also in adjacent muscle groups, as noted in the studies by Caliandro P. et al. (2012) [17] and Toscano M. et al. (2019) [16]. However, the magnitude of spasticity reduction varied between studies, likely due to differences in vibration parameters (e.g., amplitude, frequency) and the muscle groups targeted. The study by Caliandro P. et al. (2012) [17], for instance, did not observe significant changes in MAS scores, which may be attributed to the chronic stage of spasticity in their patient population. This raises the question of whether focal vibration is more effective in the subacute phase of stroke rehabilitation, where neural plasticity is more robust, as suggested by Toscano M. et al. (2019) [16]. Motor recovery was another key outcome in this review, with most studies reporting improvements in functional measures such as the Wolf Motor Function Test (WMFT) and the Fugl-Meyer Assessment (FMA). The study by Celletti C. et al. (2017) [15] highlighted the synergistic effect of combining focal vibration with progressive modular rebalancing (RMP), where patients exhibited superior motor recovery compared to traditional rehabilitation alone. Similarly, Calabrò R.S. et al. (2017) [19] found that the combination of focal vibration and robotic rehabilitation yielded significant improvements in motor performance, suggesting that focal vibration enhances the efficacy of task-specific exercises by modulating neuromuscular control. The mechanisms underpinning these improvements may lie in the modulation of cortico-spinal excitability. Calabrò et al. (2017) demonstrated a significant reduction in short-interval cortical inhibition (SICI) and Hoffman’s Reflex (HMR), which indicates that focal vibration therapy influences both cortical and spinal circuits. This neuromodulatory effect likely facilitates motor relearning by increasing the excitability of motor pathways and promoting neuroplasticity. However, despite these promising results, it remains unclear whether these improvements are sustained over the long term, as most studies in this review only assessed short-term outcomes. Future research should focus on the long-term effects of focal vibration on motor recovery to determine if the initial gains translate into lasting functional improvements. The reduction of pain, as observed in Costantino C. et al. (2017) [18] and Celletti C. et al. (2017) [15], adds an additional layer of benefit to the use of focal vibration therapy. Both studies reported significant decreases in pain scores, as measured by the Visual Analog Scale (VAS) [49] and the Numerical Rating Scale (NRS) [50], which can be attributed to the neuromodulatory effects of vibration on pain pathways. Focal vibration is known to stimulate afferent fibers and inhibit nociceptive inputs, thereby reducing pain perception. This analgesic effect is particularly relevant for post-stroke patients, as chronic pain can severely limit engagement in rehabilitation activities [49,50]. However, the mechanisms behind pain reduction are not fully elucidated, and further studies are required to explore the specific neural circuits involved. While this review indicates that focal vibration therapy shows significant potential in post-stroke rehabilitation, it is crucial to acknowledge the current limitations in its application. The existing studies show considerable variability in key intervention parameters, including vibration frequency, amplitude, duration, and the specific muscle groups targeted. This inconsistency underscores the fact that, at present, there are no well-defined, evidence-based standards for these parameters, making it difficult to implement focal vibration therapy consistently in clinical practice. This gap highlights the need for future research to rigorously investigate and establish optimal values for each parameter tailored to the specific stages of stroke recovery and individual patient characteristics. It is essential for the field to move beyond excessive caution and face the current state of uncertainty boldly, recognizing that the method remains unstandardized and thus inconsistent in its effects. Second, most studies in this review only assessed short-term outcomes, typically within a few weeks of the intervention. The long-term effects of focal vibration therapy, particularly its ability to sustain improvements in motor function and spasticity reduction, remain unclear. Longitudinal studies with extended follow-up periods are needed to determine whether these short-term gains persist and translate into lasting functional benefits. Third, several of the studies, such as those by Toscano M. et al. (2019) [16] and Costantino C. et al. (2017) [18], had relatively small sample sizes, which may limit the generalizability of their findings. The findings from the six RCTs reviewed indicate that focal vibration therapy can effectively reduce spasticity, particularly when applied during the subacute phase, with improvements reported in up to 65% of patients. Additionally, when combined with other rehabilitation approaches, such as robotic-assisted exercises or motor relearning programs, focal vibration therapy demonstrated enhanced motor function recovery in 70% of cases compared to standard therapy alone. This suggests a synergistic effect that supports its integration into comprehensive rehabilitation protocols. However, standardization of treatment parameters, including frequency and duration, remains necessary to optimize outcomes. Larger, multi-center trials are needed to confirm the efficacy of focal vibration therapy and establish its clinical utility in broader stroke populations. The main limitations include the lack of standardized information on the pressure area of vibration, the force applied (kgf or N), and side effects such as the itching sensation often reported during application. Additionally, the adaptation effect, where the effectiveness diminishes after a few seconds of application, poses another challenge for standardization and long-term effectiveness. Additionally, in some studies, blinding was not adequately described, which may introduce bias in outcome assessment. Although objective measures such as MAS and WMFT were used, the subjective nature of pain and functional assessments could be influenced by participant or assessor expectations. Finally, the efficacy of focal vibration therapy may vary depending on the stage of stroke recovery. While some studies focused on chronic stroke patients, others included participants in the subacute phase. It is possible that focal vibration is more effective in earlier stages of recovery when neuroplasticity is more prominent. This highlights the need for stratified research that investigates the timing of intervention relative to stroke onset. Focal mechanical vibration therapy appears to offer a promising adjunct to traditional rehabilitation in post-stroke patients, particularly in reducing spasticity, enhancing motor recovery, and managing pain. However, the current body of evidence is limited by heterogeneity in study designs, short-term follow-up, and small sample sizes. Future research should focus on establishing standardized protocols, exploring long-term outcomes, and conducting large-scale trials to better understand the full potential of this therapy. Despite these limitations, focal vibration therapy could play an important role in optimizing functional outcomes and improving the quality of life for stroke survivors.

### Clinical Practice Implications

Focal mechanical vibration therapy offers a complementary intervention for managing post-stroke spasticity, improving motor function, and reducing pain. Its non-invasive application can be easily integrated into existing rehabilitation protocols, particularly in combination with conventional therapies like motor relearning or robotic-assisted exercises. Clinical evidence suggests that it may be most beneficial in the subacute phase of stroke recovery. However, further research is needed to establish standardized treatment parameters and long-term efficacy. Its practical use may be best suited for targeted neuromuscular stimulation in both inpatient and outpatient rehabilitation settings.

## 5. Conclusions

Focal mechanical vibration therapy (FMVT) shows potential as an effective adjunct in post-stroke rehabilitation, particularly for reducing spasticity, enhancing motor recovery, and managing pain. The therapy’s non-invasive nature and ease of integration into clinical practice make it a promising tool for enhancing rehabilitation outcomes. However, the current evidence is limited by the heterogeneity in study designs, small sample sizes, and the short-term focus of most trials. Despite these promising aspects, there are significant disadvantages and challenges associated with FMVT. The lack of standardized parameters, such as vibration frequency, amplitude, duration, and specific muscle targeting, makes consistent clinical implementation difficult. Additionally, side effects such as itching sensations during treatment and the adaptation effect, where the therapy’s effectiveness diminishes after prolonged application, pose further complications. Future research should prioritize establishing evidence-based standards for the application parameters of FMVT, explore long-term outcomes through longitudinal studies, and conduct large-scale trials to determine its full clinical utility. Only through addressing these limitations can FMVT be reliably integrated into standard post-stroke rehabilitation protocols and improve long-term patient outcomes.

## Figures and Tables

**Table 1:** Detailed Characteristics of Studies Evaluating Focal Mechanical Vibration Therapy in Post-Stroke Patients.

Title, Author, Year of Publication, Study Type	Inclusion and Exclusion Criteria, No. of Participants	Intervention	Outcomes	Results
Focal vibration of the plantarflexor and dorsiflexor muscles improves post-stroke spasticity: a randomized single-blind controlled trial Chen Y.L. et al. (2022) RCT [45]	Inclusion: —First ischemic or hemorrhagic stroke diagnosis—Age 25–80 years—Stroke onset between 1 month and 2 years prior—MAS score between 1+ and 3—Able to follow verbal commands and sign consent forms—Able to lie prone/supine for 30 min. Exclusion:—Ankle muscle contracture on the affected side—Peripheral neuropathy—Changes in medication for spasticity or botulinum injections < 3 months prior—Contraindications for focal vibration—Participation in other clinical trials—MMSE < 18.	15 daily sessions of 30 min each CON group: Bobath therapy and motor relearning. FV_GM and FV_TA groups: Focal vibration at 3 mm amplitude and 40 Hz frequency added to conventional protocol (30 min therapy +20 min vibration).	Primary outcome: Reduction in spasticity (MAS). Secondary outcomes: Reduction in ankle clonus (Clonus Test), improvement in walking function (FAC).	Significant difference found in remission rates for MAS and Clonus Test scores in the vibration groups compared to the control group. The control group showed better improvement in walking capacity, while the FV_GM group demonstrated reduced gastrocnemius rigidity, spasticity, and ankle clonus. Total participants: 69.
Focal Muscle Vibration and Progressive Modular Rebalancing with neurokinetic facilitations in post-stroke recovery of the upper limb Celletti C. et al. (2017) RCT [15]	Inclusion: —First ischemic or hemorrhagic stroke diagnosis—Stroke onset at least one year prior. Exclusion: —Significant cardiovascular complications—Peripheral arterial disease—Cognitive deficits preventing simple commands—Prior treatments with FMV and RMP. Participants included: 18.	Two weekly sessions of one hour over 6 weeks. FMV + RMP group: Focal vibration at 0.2–0.5 mm amplitude, 100 Hz frequency, for three sets of 10 min each, with 1 min rest intervals + progressive modular rebalancing exercises focusing on upper limb kinetic chains. RMP + CP group: RMP exercises + traditional physiotherapy. CP group: Traditional physiotherapy.	Primary outcome: Upper limb functionality (WMFT). Secondary outcomes: Reduction in spasticity (MAS), reduction in pain (VAS), increase in muscle strength (MI).	Upper limb functionality improved most in the FMV + RMP and FMV + CP groups (FMV + RMP: *p* = 0.027; FMV + CP: *p* = 0.026; CP: *p* = 0.109). Spasticity reduction observed in all groups with varying degrees of success (FMV + RMP: *p* = 0.027; FMV + CP: *p* = 0.026; CP: *p* = 0.042). Pain decreased and muscle strength increased in the FMV groups.
Short-Term Effects of Focal Muscle Vibration on Motor Recovery After Acute Stroke: A Pilot Randomized Sham-Controlled Study Toscano M. et al. (2019) RCT [16]	Inclusion: —Age > 18 years—Diagnosis of first confirmed stroke—Motor deficits in upper and/or lower limbs—Ability to contract affected muscles isometrically. Exclusion: —History of TIA—Patients with aphasia, neglect, or apraxia—Cerebral venous thrombosis. Total participants: 22.	Three sets of 10 min each, with 1 min rest intervals, for 3 consecutive days. Study group: Focal vibration at 0.2–0.5 mm amplitude, 100 Hz frequency, applied to the belly of the affected muscle in supine position, with isometric contraction. Control group: Sham treatment.	Outcomes: —Stroke severity (NIHSS)—Motor and functional improvement of limbs (Fugl-Meyer + MI)—Reduction in spasticity (MAS).	Patients treated with rMV showed significant clinical improvement compared to the control group in NIHSS (*p* < 0.001), Fugl-Meyer (*p* = 0.001), and Motricity Index (*p* < 0.001).
Focal muscle vibration in the treatment of upper limb spasticity: a pilot randomized controlled trial in patients with chronic stroke Caliandro P. et al. (2012) RCT [17]	Inclusion: —Patients with chronic spastic hemiplegia/hemiparesis—Stroke onset at least one year prior (ischemic or hemorrhagic). Exclusion: —Cardiovascular complications within the last 12 months—Upper limb botulinum injections within the last year—Surgery within the last year—MMSE < 23. Participants included: 49.	Three sets of 10 min each, with 1 min rest intervals, for 3 consecutive days. Study group: Focal vibration at 0.2–0.5 mm amplitude, 100 Hz frequency, applied to the muscle belly in supine position, with isometric contraction. Control group: Sham treatment.	Primary outcome: Upper limb functionality (WMFT). Secondary outcomes: Reduction in spasticity (MAS), reduction in pain (VAS).	Significant improvement in WMFT scores for the study group (*p* = 0.006), but no significant changes in MAS or VAS scores.
Short-term effect of local muscle vibration treatment versus sham therapy on upper limb in chronic post-stroke patients: a randomized controlled trial Costantino C. et al. (2017) RCT [18]	Inclusion: —Chronic ischemic or hemorrhagic stroke survivors—Stroke onset at least 12 months prior—Unilateral upper limb spasticity (MAS 1-4)—No cognitive impairments. Exclusion: —Participation in other treatment programs—Inflammatory joint diseases—Neoplastic diseases—Use of anticoagulants or antiepileptics—Hearing aids—Artificial cardiac pacemakers—Recent trauma—Joint prostheses—Recent botulinum toxin treatments—Metal implants. Participants included: 32.	Three weekly sessions of 30 min over 4 weeks. Study group: Focal vibration at 0.2 mm amplitude, 300 Hz frequency, applied to triceps brachii and radial wrist extensors. Control group: Sham treatment.	Primary outcome: Grip strength (Hand Grip Strength Test). Secondary outcomes: Reduction in spasticity (MAS), reduction in disability (QuickDASH, FIM, FMA-UE, JTT), reduction in pain (NRS).	Significant improvements in all measured outcomes for the study group compared to the control group. Both paretic and non-paretic hands showed improvements in the study group.
Is two better than one? Muscle vibration plus robotic rehabilitation to improve upper limb spasticity and function: A pilot randomized controlled trial Calabrò R.S. et al. (2017) RCT [19]	Inclusion: —First ischemic stroke in left hemisphere, with onset at least 3 months prior—Deficits in shoulder abductors, flexors, and elbow extensors—Spasticity in biceps brachii, pectoralis major, latissimus dorsi (MAS 1+ to 3)—Age 50–80 years—Caucasian ethnicity. Exclusion: —Neurodegenerative diseases or concurrent surgeries—Severe cognitive or language deficits—Systemic or osteo-articular conditions—Central or peripheral sensitivity impairments—Concurrent use of medications for spasticity. Participants included: 20.	Five weekly sessions of one hour over 8 weeks. Robotic rehabilitation (RR): Repetitive exercises for shoulder and elbow movements. Focal mechanical vibration (FMV): Applied to antagonist muscles during RR (triceps brachii, supraspinatus, deltoid) with amplitude set between 0.2–0.4 mm and frequency of 80 Hz.	Primary outcomes: Reduction in spasticity (MAS), reduction in cortical excitability (SICI), reduction in spinal motor circuit excitability (HMR). Secondary outcomes: Upper limb functional recovery (FMA-UE), reduction in disability (FIM), improvement in mood and anxiety (HRS-D, HRS-A).	After 8 weeks, all study group patients achieved the minimum goal of reducing MAS scores by 1 point and at least a 15% reduction in SICI and HMR values. Significant differences between groups were observed in all primary and secondary outcomes (*p* < 0.001).

Legend: FAC: Functional Ambulation Classification, FIM: Functional Independence Measure, FMV: Focal Mechanical Vibration, FMA-UE: Fugl-Meyer Assessment for Upper Extremities, HMR: Hoffman’s Reflex Measurement, HRS-D: Hamilton Rating Scale for Depression, HRS-A: Hamilton Rating Scale for Anxiety, JTT: Jebsen-Taylor Hand Function Test, MAS: Modified Ashworth Scale, MI: Motricity Index, MMSE: Mini-Mental State Examination, NIHSS: National Institutes of Health Stroke Scale, NRS: Numerical Rating Scale, QuickDASH: Disabilities of the Arm, Shoulder, and Hand (Quick form), RMP: Progressive Modular Rebalancing, SICI: Short Interval Cortical Inhibition, VAS: Visual Analog Scale, WMFT: Wolf Motor Function.

## References

[1] Sakakibara B.M., Kim A.J., Eng J.J. (2017). A Systematic Review and Meta-Analysis on Self-Management for Improving Risk Factor Control in Stroke Patients. Int. J. Behav. Med..

[2] (2012). Linee Guida SPREAD VII Edizione.

[3] Donati D., Farì G., Giorgi F., Marvulli R., Quarta F., Bernetti A., Tedeschi R. (2024). Efficacy of Motor Imagery in the Rehabilitation of Stroke Patients: A Scope Review. OBM Neurobiol..

[4] Bohannon R.W. (2006). Hand-Held Dynamometry: Adoption 1900–2005. Percept. Mot. Ski..

[5] Nicolai L., Massberg S. (2024). Myeloid Cells Take Ischemic Insult to Heart. Trends Immunol..

[6] Sarmah D., Datta A., Rana N., Suthar P., Gupta V., Kaur H., Ghosh B., Levoux J., Rodriguez A.-M., Yavagal D.R. (2024). SIRT-1/RHOT-1/PGC-1α Loop Modulates Mitochondrial Biogenesis and Transfer to Offer Resilience Following Endovascular Stem Cell Therapy in Ischemic Stroke. Free Radic. Biol. Med..

[7] Kim C.M., Eng J.J. (2003). The Relationship of Lower-Extremity Muscle Torque to Locomotor Performance in People with Stroke. Phys. Ther..

[8] Wist S., Clivaz J., Sattelmayer M. (2016). Muscle Strengthening for Hemiparesis after Stroke: A Meta-Analysis. Ann. Phys. Rehabil. Med..

[9] Pantano P., Formisano R., Ricci M., Di Piero V., Sabatini U., Barbanti P., Fiorelli M., Bozzao L., Lenzi G.L. (1995). Prolonged Muscular Flaccidity after Stroke. Morphological and Functional Brain Alterations. Brain.

[10] Tedeschi R. (2023). Kinematic and Plantar Pressure Analysis in Strumpell-Lorrain Disease: A Case Report. Brain Disord..

[11] Pandyan A.D., Gregoric M., Barnes M.P., Wood D., Van Wijck F., Burridge J., Hermens H., Johnson G.R. (2005). Spasticity: Clinical Perceptions, Neurological Realities and Meaningful Measurement. Disabil. Rehabil..

[12] Karnath H.-O. (2007). Pusher Syndrome—A Frequent but Little-Known Disturbance of Body Orientation Perception. J. Neurol..

[13] Hsu Y.-H., Chen W.-Y., Lin H.-C., Wang W.T.J., Shih Y.-F. (2009). The Effects of Taping on Scapular Kinematics and Muscle Performance in Baseball Players with Shoulder Impingement Syndrome. J. Electromyogr. Kinesiol..

[14] Chen Y.-L., Jiang L.-J., Cheng Y.-Y., Chen C., Hu J., Zhang A.-J., Hua Y., Bai Y.-L. (2023). Focal Vibration of the Plantarflexor and Dorsiflexor Muscles Improves Poststroke Spasticity: A Randomized Single-Blind Controlled Trial. Ann. Phys. Rehabil. Med..

[15] Celletti C., Sinibaldi E., Pierelli F., Monari G., Camerota F. (2017). Focal Muscle Vibration and Progressive Modular Rebalancing with Neurokinetic Facilitations in Post- Stroke Recovery of Upper Limb. Clin. Ter..

[16] Viganò A., Celletti C., Giuliani G., Jannini T.B., Marenco F., Maestrini I., Zumpano R., Vicenzini E., Altieri M., Camerota F. (2023). Focal Muscle Vibration (fMV) for Post-Stroke Motor Recovery: Multisite Neuroplasticity Induction, Timing of Intervention, Clinical Approaches, and Prospects from a Narrative Review. Vibration.

[17] Caliandro P., Celletti C., Padua L., Minciotti I., Russo G., Granata G., La Torre G., Granieri E., Camerota F. (2012). Focal Muscle Vibration in the Treatment of Upper Limb Spasticity: A Pilot Randomized Controlled Trial in Patients with Chronic Stroke. Arch. Phys. Med. Rehabil..

[18] Costantino C., Galuppo L., Romiti D. (2017). Short-Term Effect of Local Muscle Vibration Treatment versus Sham Therapy on Upper Limb in Chronic Post-Stroke Patients: A Randomized Controlled Trial. Eur. J. Phys. Rehabil. Med..

[19] Calabrò R.S., Naro A., Russo M., Milardi D., Leo A., Filoni S., Trinchera A., Bramanti P. (2017). Is Two Better than One? Muscle Vibration plus Robotic Rehabilitation to Improve Upper Limb Spasticity and Function: A Pilot Randomized Controlled Trial. PLoS ONE.

[20] Tedeschi R. (2024). Assessing the Impact of Novafon Local Vibration Voice Therapy on Voice Disorders: A Comprehensive Review. Rwanda Med. J..

[21] Jones C.A., Colletti C.M., Ding M.-C. (2020). Post-Stroke Dysphagia: Recent Insights and Unanswered Questions. Curr. Neurol. Neurosci. Rep..

[22] Grossman M., Irwin D.J. (2018). Primary Progressive Aphasia and Stroke Aphasia. Continuum.

[23] Barrett A.M. (2021). Spatial Neglect and Anosognosia After Right Brain Stroke. Continuum.

[24] Greene J.D.W. (2005). Apraxia, Agnosias, and Higher Visual Function Abnormalities. J. Neurol. Neurosurg. Psychiatry.

[25] Chiaramonte R., Vecchio M. (2021). Rehabilitation of Focal Hand Dystonia in Musicians: A Systematic Review of the Studies. Rev. Neurol..

[26] Sarwar A., Emmady P.D. (2024). Spatial Neglect. StatPearls.

[27] Pekna M., Pekny M., Nilsson M. (2012). Modulation of Neural Plasticity as a Basis for Stroke Rehabilitation. Stroke.

[28] Puderbaugh M., Emmady P.D. (2024). Neuroplasticity. StatPearls.

[29] Sophie Su Y., Veeravagu A., Grant G., Laskowitz D., Grant G. (2016). Neuroplasticity after Traumatic Brain Injury. Translational Research in Traumatic Brain Injury.

[30] van Nes I.J.W., Latour H., Schils F., Meijer R., van Kuijk A., Geurts A.C.H. (2006). Long-Term Effects of 6-Week Whole-Body Vibration on Balance Recovery and Activities of Daily Living in the Postacute Phase of Stroke: A Randomized, Controlled Trial. Stroke.

[31] Murillo N., Valls-Sole J., Vidal J., Opisso E., Medina J., Kumru H. (2014). Focal Vibration in Neurorehabilitation. Eur. J. Phys. Rehabil. Med..

[32] Roll J.P., Vedel J.P. (1982). Kinaesthetic Role of Muscle Afferents in Man, Studied by Tendon Vibration and Microneurography. Exp. Brain Res..

[33] Roll J.P., Vedel J.P., Ribot E. (1989). Alteration of Proprioceptive Messages Induced by Tendon Vibration in Man: A Microneurographic Study. Exp. Brain Res..

[34] Tedeschi R., Amoruso V., Boetto V., Glorioso D., D’Auria L., Donati D. (2024). COVID-19-Associated Cerebellitis: A Case Report and Rehabilitation Outcome. Cerebellum.

[35] Rosenkranz K., Rothwell J.C. (2003). Differential Effect of Muscle Vibration on Intracortical Inhibitory Circuits in Humans. J. Physiol..

[36] Fattorini L., Ferraresi A., Rodio A., Azzena G. (2006). Motor Performance Changes Induced by Muscle Vibration. Eur. J. Appl. Physiol..

[37] Vojinovic T.J., Linley E., Zivanovic A., Rui Loureiro C.V. (2019). Effects of Focal Vibration and Robotic Assistive Therapy on Upper Limb Spasticity in Incomplete Spinal Cord Injury. IEEE Int. Conf. Rehabil. Robot.

[38] Benedetti M.G., Boccia G., Cavazzuti L., Magnani E., Mariani E., Rainoldi A., Casale R. (2017). Localized Muscle Vibration Reverses Quadriceps Muscle Hypotrophy and Improves Physical Function: A Clinical and Electrophysiological Study. Int. J. Rehabil. Res..

[39] Tedeschi R., Platano D., Donati D., Giorgi F. (2024). Integrating the Drucebo Effect into PM&R: Enhancing Outcomes through Expectation Management. Am. J. Phys. Med. Rehabil..

[40] Picelli A., DI Censo R., Angeli C., Spina S., Santamato A., Baricich A., Smania N., Filippetti M. (2024). Is the Silfverskiöld Test a Valid Tool for Evaluating Calf Muscles Spastic Overactivity in Patients with Stroke? A Retrospective Observational Study. Eur. J. Phys. Rehabil. Med..

[41] Facciorusso S., Guanziroli E., Brambilla C., Spina S., Giraud M., Molinari Tosatti L., Santamato A., Molteni F., Scano A. (2024). Muscle Synergies in Upper Limb Stroke Rehabilitation: A Scoping Review. Eur. J. Phys. Rehabil. Med..

[42] Tedeschi R. (2023). Unveiling the Potential of Trigger Point Therapy: Exploring Its Efficacy in Managing Muscular Spasticity—A Scoping Review. Muscles Ligaments Tendons J..

[43] Casadei I., Betti F., Tedeschi R. (2023). Assessment of Muscle Tone in Patients with Acquired Brain Injury: A Systematic Review. Motricite Cerebrale.

[44] Tedeschi R. (2024). Reevaluating the Drucebo Effect: Implications for Physiotherapy Practice. J. Psychosoc. Rehabil. Ment. Health.

[45] Chang W.-D., Chen S., Tsou Y.-A. (2021). Effects of Whole-Body Vibration and Balance Training on Female Athletes with Chronic Ankle Instability. J. Clin. Med..

[46] Tedeschi R. (2023). Biomechanical Alterations in Lower Limb Lymphedema: Implications for Walking Ability and Rehabilitation. Phlebology.

[47] Peters: Joanna Briggs Institute Reviewer’s Manual, JBI—Google Scholar. https://scholar-google-com.ezproxy.unibo.it/scholar_lookup?hl=en&publication_year=2020&author=MDJ+Peters&author=C+Godfrey&author=P+McInerney&author=Z+Munn&author=AC+Tricco&author=H+Khalil&title=Joanna+Briggs+Institute+Reviewer%27s+Manual%2C+JBI.

[48] Page M.J., McKenzie J.E., Bossuyt P.M., Boutron I., Hoffmann T.C., Mulrow C.D., Shamseer L., Tetzlaff J.M., Akl E.A., Brennan S.E. (2021). The PRISMA 2020 Statement: An Updated Guideline for Reporting Systematic Reviews. BMJ.

[49] Delgado D.A., Lambert B.S., Boutris N., McCulloch P.C., Robbins A.B., Moreno M.R., Harris J.D. (2018). Validation of Digital Visual Analog Scale Pain Scoring With a Traditional Paper-Based Visual Analog Scale in Adults. J. Am. Acad. Orthop. Surg. Glob. Res. Rev..

[50] Farrar J.T., Young J.P., LaMoreaux L., Werth J.L., Poole M.R. (2001). Clinical Importance of Changes in Chronic Pain Intensity Measured on an 11-Point Numerical Pain Rating Scale. Pain.

